# Genome-wide association study identifies two loci influencing plasma neurofilament light levels

**DOI:** 10.1186/s12920-018-0364-8

**Published:** 2018-05-10

**Authors:** Jie-Qiong Li, Xiang-Zhen Yuan, Hai-Yan Li, Xi-Peng Cao, Jin-Tai Yu, Lan Tan, Wei-An Chen, Alzheimer’s Disease Neuroimaging Initiative

**Affiliations:** 1 Department of Neurology, Qingdao Municipal Hospital, Qingdao University, No.5 Donghai Middle Road, Qingdao, 266071 Shandong Province China; 2 Department of Neurology, Weihaiwei People’s Hospital, Weihai, China; 3 Clinical Research Center, Qingdao Municipal Hospital, Qingdao University, Qingdao, China; 4 Department of Neurology, The First Affiliated Hospital of Wenzhou Medical University, Nanbaixiang Road, Wenzhou, 325000 Zhejiang Province China

**Keywords:** Genome-wide association study, Plasma NFL, Alzheimer disease, *LUZP2*, *GABRB2*, Genetic factors

## Abstract

**Background:**

Plasma neurofilament light (NFL) is a promising biomarker for Alzheimer disease (AD), which increases in the early stage of AD and is associated with the progression of AD. We performed a genome-wide association study (GWAS) of plasma NFL in Alzheimer’s Disease Neuroimaging Initiative 1 (ADNI-1) cohort to identify novel variants associated with AD.

**Methods:**

This study included 179 cognitively healthy controls (HC), 176 patients with mild cognitive impairment (MCI), and 172 patients with AD. All subjects were restricted to non-Hispanic Caucasian derived from the ADNI cohort and met all quality control (QC) criteria. Association of plasma NFL with the genetic variants was assessed using PLINK with an additive genetic model, i.e.dose-dependent effect of the minor alleles. The influence of a genetic variant associated with plasma NFL (rs7943454) on brain structure was further assessed using PLINK with a linear regression model.

**Results:**

The minor allele (T) of rs7943454 in leucine zipper protein 2 gene (*LUZP2*) was associated with higher plasma NFL at suggestive levels (*P* = 1.39 × 10^− 6^) in a dose-dependent fashion. In contrast, the minor allele (G) of rs640476 near *GABRB2* was associated with lower plasma NFL at suggestive levels (*P* = 6.71 × 10^− 6^) in a dose-dependent effect in all diagnostic groups except the MCI group. Furthermore, the minor allele (T) of rs7943454 within *LUZP2* increased the onset risk of AD (odds ratio = 1.547, confidence interval 95% = 1.018–2.351) and was associated with atrophy of right middle temporal gyrus in the whole cohort in the longitudinal study (*P* = 0.0234).

**Conclusion:**

GWAS found the associations of two single nucleotide polymorphisms (rs7943454 and rs640476) with plasma NFL at suggestive levels. Rs7943454 in *LUZP2* was associated with the onset risk of AD and atrophy of right middle temporal gyrusin the whole cohort. Using an endophenotype-based approach, we identified rs7943454 as a new AD risk locus.

**Electronic supplementary material:**

The online version of this article (10.1186/s12920-018-0364-8) contains supplementary material, which is available to authorized users.

## Background

Alzheimer disease (AD) is the main cause of dementia and one of the major challenges for health care across the world, which is characterized pathologically by extracellular accumulation of amyloid-β (Aβ), intracellular deposition of neurofibrillary tangles (NFT), neuronal loss and synaptic dysfunction [1]. Well-established cerebrospinal fluid (CSF) biomarkers including Aβ_42_, total-tau (t-tau), phosphorylated tau (p-tau) have been used for the diagnosis of AD and monitoring its progression [2], but the their application is hampered by a high degree of invasiveness, complex operations and high costs. Biomarkers in peripheral blood are more appropriate screening tools for AD among old individuals to monitor AD progression. Interestingly, recent studies using ultra-sensitive assay showed that plasma neurofilament light (NFL), the main component of neurofilaments (cytoskeletal protein of neurons), increased in patients with AD dementia and was associated with other established CSF and neuroimaging biomarkers of AD [3, 4]. Plasma NFL is a noninvasive biomarker for neuronal injury in AD compared with CSF biomarkers. Thus, it has the potential for monitoring AD progression [3].

AD is a clinically heterogeneous neurodegenerative disease with a strong genetic component. Genetic risk factors of AD impact the CSF or neuroimaging biomarkers through which they might modulate the process of AD [5]. Thus, biomarkers for AD may be used as endophenotypes to explore the genetic factors that impact their metabolism [6–8]. Based on the association between plasma NFL and AD, we performed a genome-wide association study (GWAS) using plasma NFL as an endophenotype of AD to explore genetic factors involved in plasma NFL metabolism. We hypothesized that these genetic factors may influence pathological change in AD.

## Methods

### Subjects

In this study, 172 AD patients, 176 subjects with mild cognitive impairment (MCI), and 179 healthy controls (HC) whose data met all quality control (QC) criteria were included from the Alzheimer’s Disease Neuroimaging Initiative 1 (ADNI-1) cohort. The full cohort with plasma NFL and genotype data included 578 subjects. All the subjects were restricted to non-Hispanic Caucasian checked with their pedigree information checked to reduce potential bias of population stratification that might confound GWAS results. This step removed 40 subjects. After QC of the plasma NFL levels and removal of 11 outliers, there were 527 subjects with plasma NFL data left. The detailed demographic information and plasma NFL data have been shown in Table 1.

**Table 1:** The demographic information of participants with plasma NFL data

Baseline diagnosis	AD	MCI	HC	Total
*n*	172	176	179	527
Age (years), mean ± SD (range)	76 ± 7 (56–91)	75 ± 8 (54–89)	76 ± 5 (62–90)	75 ± 7 (54–91)
Gender, male/female	90/82	117/59	103/76	310/217
APOE ε4 carrier (%)	66.9	52.8	26.8	48.6
Plasma NFL (pg/ml), mean ± SD^a^	48.7 ± 20.9	39.9 ± 17.7	32.8 ± 15.5	40.4 ± 19.3

*Abbreviations: AD* Alzheimer’s disease, *APOE* Apolipoprotein E; *HC* healthy control, *MCI* mild cognitive impairment, *NFL* neurofilament light, *SD* standard deviation

^a^Plasma NFL levels were different across the 3 diagnostic groups (*P* < 0.0001). Tukey’s multiple comparisons test showed that AD patients had higher plasma NFL levels compared with MCI group and healthy controls (*P* < 0.001). MCI group also had higher plasma NFL levels compared to healthy controls (*P* = 0.0007)

### ADNI dataset

ADNI was launched in 2003 by the National Institute on Aging, the National Institute of Biomedical Imaging and Bioengineering, the Food and Drug Administration, private pharmaceutical companies and nonprofit organizations. ADNI was established to develop serial magnetic resonance imaging (MRI), positron emission tomography (PET), and a combination of biomarkers, neuropsychological and clinical assessment to improve early diagnosis and measure the progression of AD [9]. The ADNI database has three protocols (ADNI 1, ADNI 2 and ADNI Grand Opportunities (ADNI GO)) at present and recruited more than 1500 participants including normal older subjects, MCI and early AD in this research. More information is available on the website of ADNI (www.loni.ucla.edu/ADNI).

### Plasma measurements and quality control

Plasma NFL was analyzed using the ultrasensitive Single Molecule array (Simoa) technique as previously described [10]. The assay used a combination of monoclonal antibodies and purified bovine NFL as a calibrator. Analytical sensitivity was < 1.0 pg/mL, and the NFL levels in all tested sample were above the detection limit. Further QC was performed to reduce the potential influence of extreme outliers on statistical results. Mean and standard deviations (SD) of baseline plasma NFL were calculated. Subjects who had a value which is 3-fold SD greater or smaller than the mean value (< 42.8–3 × 26.8 pg/mL or > 42.8 + 3 × 26.8 pg/mL) were removed from the analysis. This step removed 11 subjects.

### Genotyping and quality control

The ADNI 1 samples involved in this study were genotyped by Human 610-Quad BeadChip (Illumina, Inc., San Diego, CA). PLINK software (version 1.07) was used to explore the association of plasma NFL with the genetic variants using the following stringent criteria: minimum call rate for single nucleotide polymorphisms (SNPs) and individuals > 98%; minimum minor allele frequencies (MAF) > 0.20, Hardy-Weinberg equilibrium test *P* > 0.001. The restriction to SNPs with a minor allele frequency > 0.20 served to reduce the potential for false-positive results and enhance statistical power. An apolipoprotein E *(APOE)* genotyping kit was used to identify *APOE* alleles, which were defined by rs7412 and rs429358 [7].

### Brain structure on MRI

The data of MRI brain structure were derived from UCSF FreeSurfer datasets, which were used to conduct association test of rs7943454in leucine zipper protein2 gene (*LUZP2*) with brain structure. The cerebral image segmentation and analysis were performed with the FreeSurfer version5.1 (http://surfer.nmr.mgh.harvard.edu/) based on the2010 Desikan-Killanyatlas [11]. The technical details of these procedures have been described in prior publications [12]. Brian regions have been reported to be closely associated with AD such as hippocampus, parahippocampus, middle temporal gyrus, posterior cingulate, precuneus and entorhinal cortex, which were selected as our regions of interest (ROI) to analyze their its associations with rs7943454.

### Statistical analyses

Association studies of plasma NFL with the genetic variants were performed using PLINK (version 1.07) with the additive genetic model, i.e. dose-dependent effect of the minor allele. The analysis included a total of 30,1687 genotyped variants. To adjust for multiple testing, Bonferroni correction was applied and SNPs with corrected *p* < 0.01 (uncorrected *p* < 3.31 × 10–8, i.e., 0.01/301687 markers) were considered genome-wide significant. And we secondarily examined SNPs with uncorrected *p* values less than 10–5 to identify potential candidates. Age, gender and diagnosis were included as covariates. Bonferroni correction of the *P* values by the total number of acceptable quality SNPs was used for multiple test correction. Differences in continuous variables (plasma NFL levels, volume of regional brain) were examined using one-way analysis of variance (ANOVA), and Tukey’s multiple comparisons test was used to perform the pairwise analysis after ANOVA. Genome-wide associations were visualized using a software program (R, version 3.4.0; The R Foundation). Regional associations were visualized with the Locus Zoom web tool (http://locuszoom.org/). Moreover, a multiple linear regression model was applied using PLINK to estimate coefficients for testing a possible correlation between brain structure and rs7943454. Age, gender, education, and APOE ε4status were used as covariates.

## Results

### Characteristics of included subjects

The information about these included subjects has been shown in Table 1. Briefly, 172 AD (82 women, 76 ± 7 years), 176 MCI (59 women, 75 ± 8 years) and 179 HC (76 women, 76 ± 5 years) subjects were recruited in this study. AD group had the highest frequency of ε4 allele within *APOE* gene (66.9%). AD patients (48.7 ± 20.9 pg/ml) had higher plasma NFL levels compared with MCI group (39.9 ± 17.7 pg/ml) and HC group (32.8 ± 15.5 pg/ml) (*P* < 0.001). MCI group also had higher plasma NFL levels compared to HC group (*P* = 0.0007). The sensitivity and specificity of plasma NFL used for the diagnosis of AD were 0.73 and 0.84 respectively. The area under the curve (AUC) of the model containing plasma NFL, age at baseline, gender, educational level and APOE ε4 genotype was 0.86 in predicting the onset of AD among HC controls group. By comparison, the AUROCs were 0.84 to 0.87 for CSF Aβ42, CSF t-tau, and CSF p-tau (Additional file 1: Figure S1).

### SNPs associated with plasma NFL levels

**Fig. 1:**
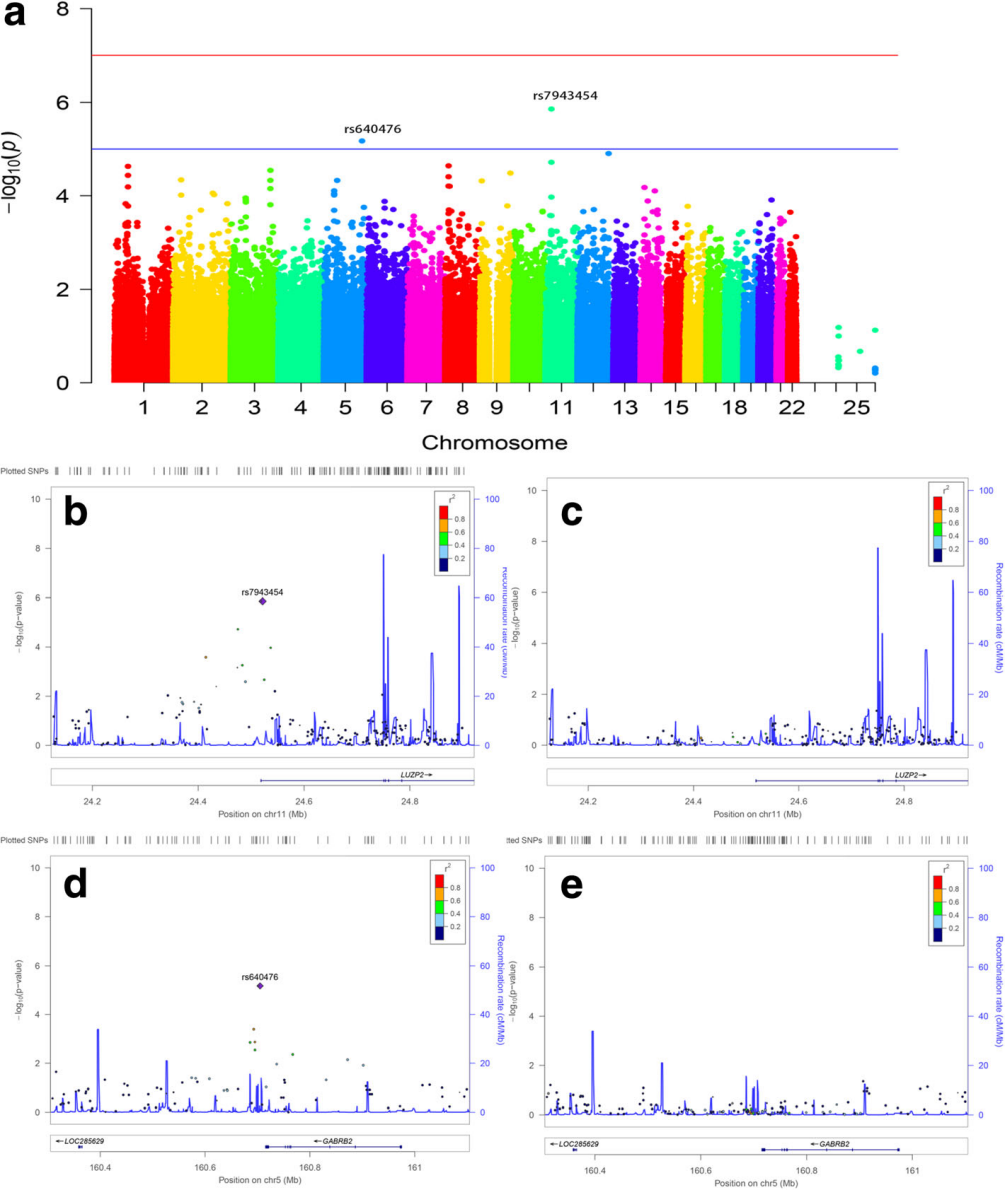
Manhattan and regional plots for associations with plasma NFL levels. **a** Genome-wide signal intensity (Manhattan) plots showing the –log_10_ (*p*-value) for individual SNPs. **b** Regional association results for the 24.2 Mb to 24.8 Mb region of chromosome 11. **c** Association results for 24.2 Mb to 24.8 Mb region of chromosome 11 controlling for rs7943454. **d** Regional association results for the 160.4 Mb to 161 Mb regions of chromosome 5. **e** Association results for 160.4 Mb to 161 Mb regions of chromosome 5 controlling for rs640476

There were 527 individuals with plasma NFL data as mentioned above. After adjusting for age, gender and diagnosis, two SNPs (rs7943454, rs640476) were identified associated with plasma NFL at suggestive levels of *P* < 10^− 5^ (Fig. 1a, Table 2). No SNPs with genome-wide significant association with plasma NFL levels was identified in this study.

**Table 2:** Top SNPs associated with plasma NFL levels (*P* values < 10^− 5^)

CHR	SNP	MAF	Closest Gene	SNP Type/Location	*P* values
11	rs7943454	0.460	*LUZP2*	intron	1.39 × 10^−6^
5	rs640476	0.297	*GABRB2*	intergenic	6.71 × 10^−6^

*Abbreviations: CHR* chromosome, *LUZP2* leucine zipper protein 2, *MAF* minor allele frequency, *SNP* single nucleotide polymorphism, *GABRB2* gamma-aminobutyric acid type A receptor beta2 subunit

**Fig. 2:**
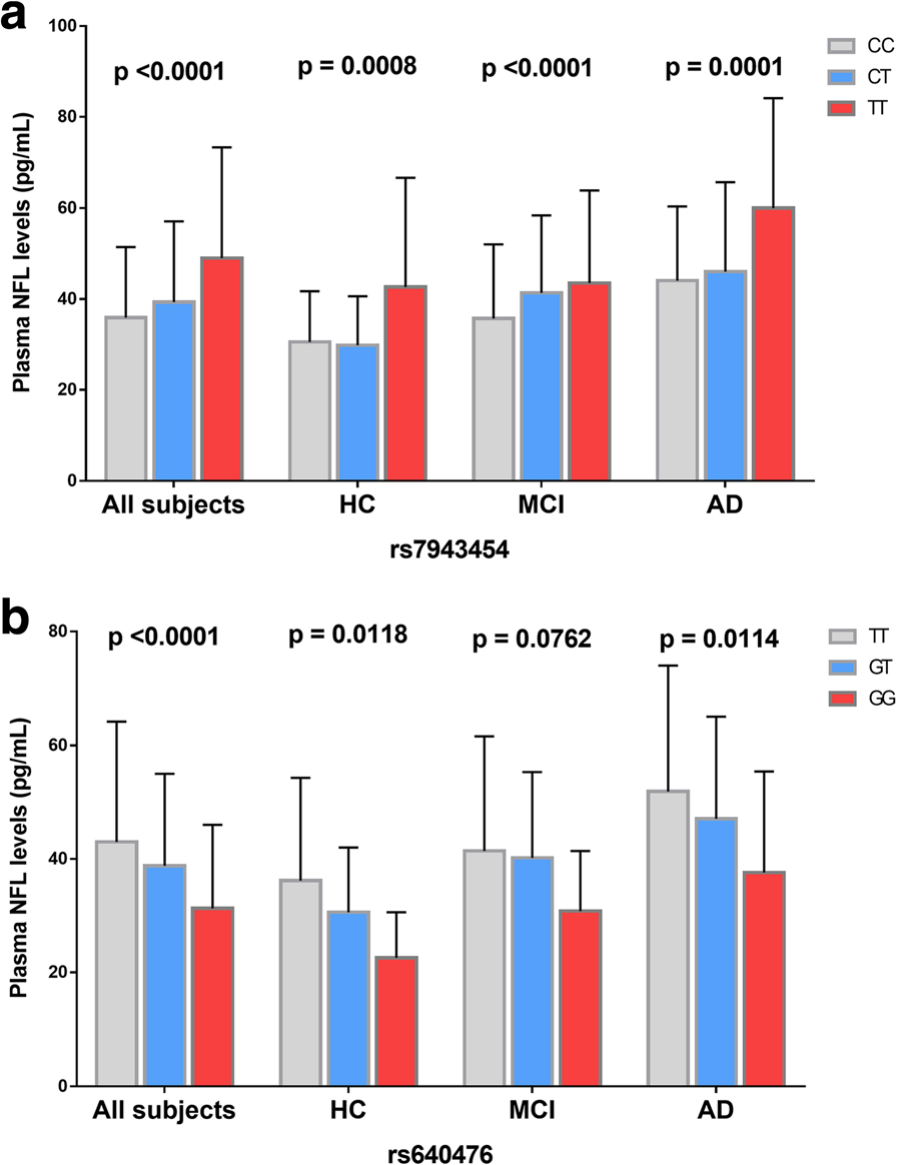
Mean plasma NFL levels of different diagnostic groups and genotypes. Mean and standard errors of plasma NFL levels are shown for groups defined by baseline diagnosis and genotypes. *P* < 0.05 was considered statistically significant after examination with a multiple linear regression model using age, gender and the diagnosis as covariates. **a** The minor alleleofrs7943454 (T) showed association with higher plasma NFL levels in a dose-dependent effect in all diagnostic groups. **b** The minor allele of rs640476 (G) showed association with lower plasma NFL levels in a dose-dependent effect in all diagnostic groups except the MCI group

The minor allele of rs7943454 (T) was associated with higher plasma NFL levels in a dose-dependent effect in all diagnostic groups (Fig. 2a). In contrast, the minor allele of rs640476 (G) showed association with lower plasma NFL levels in a dose-dependent effect in all diagnostic groups except the MCI group (Fig. 2b).

SNPs mapped closely to the two suggestive SNPs (rs7943454, rs640476) regions were also analyzed. These nearby SNPs showed association with plasma NFL levels at *P* levels lower than 0.01. However, these SNPs associated with plasma NFL also disappeared after controlling the genotype of the two suggestive SNPs (Fig. 1b and c, Fig. 1d and e). The results indicated that these nearby SNPs were driven by the two suggestive SNPs.

### Rs7943454 and onset risk of AD

The International Genomics of Alzheimer’s Disease Project (IGAP) is the largest genetic epidemiology investigation of AD risk to date. In 2013, the IGAP reported a grand-scale meta-analysis and identified 11 new susceptibility loci for AD [13]. The IGAP research was divided into a discovery step (stage 1) and a replication step (stage 2). We checked the two loci associated with plasma NFL in IGAP database in the stage 1 meta-analysis and identified rs7943454 as a risk locus for AD (*P* = 0.03476). The minor allele of rs7943454 (T) increased the onset risk of AD 1.547-fold in our analysis (odds ratio = 1.547, confidence interval 95% = 1.018–2.351).

### Impact of rs7943454 on brain structure

**Fig. 3:**
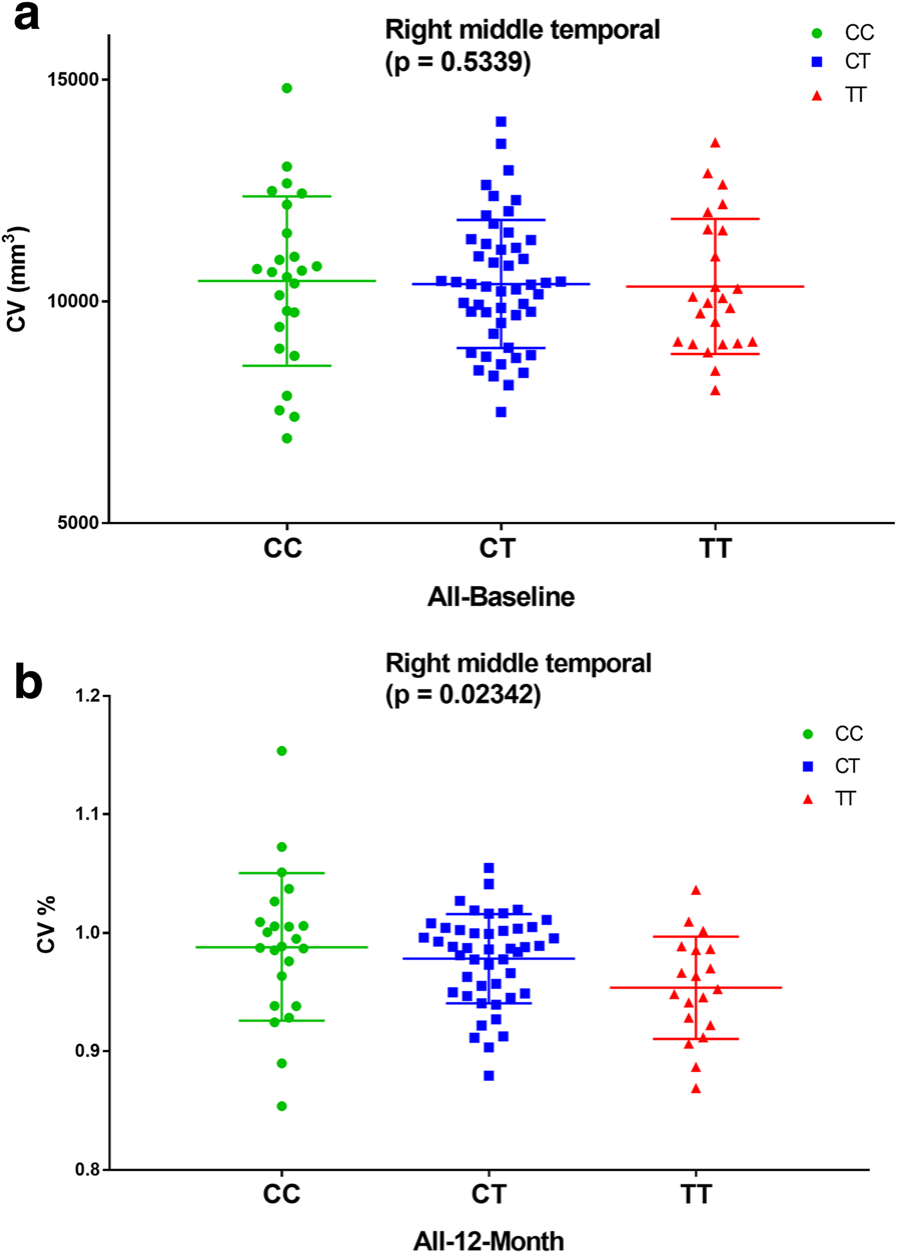
Rs7943454 and right middle temporal gyrus. **a** The volume of right middle temporal gyrus was not associated with rs7943454 at baseline in the whole cohort (*P* = 0.5339). **b** Rs7943454 increase the percentage of atrophy of right middle temporal gyrus in the whole cohort in the following-up research of 1 year (*P* = 0.0234). Subjects with TT genotypes had greater atrophy rate of right middle temporal gyrus than those with CC genotypes (*P* = 0.05)

Several cortical areas including middle temporal gyrus, posterior cingulate, precuneus, parahippocampal gyrus, and hippocampus were chosen as the ROI of the AD related MRI measures analysis. We analyzed the association of rs7943454 with AD related brain structures in a linear model using age, gender, education years, *APOE ε4* status and intracranial volume (ICV) as covariates. There was no regional cortical volume associated with rs7943454 at baseline in the hybrid population (AD, MCI, and HC subjects) (Fig. 3a). However, rs7943454 increased the percentage of atrophy of right middle temporal gyrus in the hybrid population in the one-year follow-up research (*P* = 0.0234, Fig. 3b). Subjects with TT genotype had greater atrophy rate of right middle temporal gyrus than those with CC genotype (CC: 0.9880 ± 0.0622, CT: 0.9783 ± 0.0377, TT: 0.9537 ± 0.0433) (*P* = 0.05). There was no significant difference in the volume of right middle temporal gyrus between CT genotype and CC genotype or TT genotype.

## Discussion

To our knowledge, this study was the first one using plasma NFL as an endophenotype of AD for GWAS. The use of quantitative traits in GWAS has been shown to increase statistical power over case-control designs [8]. We identified two SNPs (rs7943454, rs640476) associated with plasma NFL at suggestive levels. The minor allele (T) of rs7943454 within *LUZP2* increased the onset risk of AD and was associated with atrophy of right middle temporal gyrus in the entire cohort of the one-year longitudinal study. The use of plasma NFL as an endophenotype of AD for GWAS enabled us to identify a novel AD candidate gene in addition to examining the influence of well-known AD genes on AD biomarkers. We also found the minor allele (T) of rs7943454 in *LUZP2* was associated with higher plasma NFL levels in a dose-dependent fashion. In contrast, the minor allele (G) of rs640476 near *GABRB2* was associated with lower plasma NFL at suggestive levels in a dose-dependent effect in all diagnostic groups except the MCI group.

Plasma NFL showed significant increase in AD patients than in MCI and healthy controls (*P* < 0.001). Similar with CSF NFL, plasma is not disease-specific and even more marked increases are found in several other neurodegenerative disorders. Increasing evidence indicates that plasma NFL is a potential biomarker for the progression of AD but not for the diagnosis [4, 14–16]. Plasma NFL is noninvasive and has diagnostic accuracy for AD in the same range as established CSF biomarkers [3]. Plasma NFL may be widely used as a biomarker in clinical studies and drug development of AD, although there is still a long way to go. The elevated NFL levels in plasma also indicate that the degeneration of large-caliber axons plays an important role in the progression of AD [14].

The variation rs7943454 is located on chromosome 11p14.3 within *LUZP2* region. *LUZP2* is a leucine zipper protein coding gene that has been reported to be deleted in some patients with Wilms tumor-Aniridia-Genitourinary anomalies-mental Retardation (WAGR) syndrome [17]. It has also been reported that *LUZP2* was associated with prostate cancer and hypereosinophilic syndrome [18, 19]. The function of *LUZP2* is still unclear and inhibition of its expression did not show any obvious abnormal phenotypes in mice [20]. Rs7943454 in *LUZP2* has been reported as a risk locus of AD in IGAP, which has been validated in our analysis. The minor allele (T) of rs7943454 increased plasma NFL levels in a dose-dependent fashion and was associated with atrophy of right middle temporal gyrus in the hybrid population. Neuroimaging changes occur years before cognitive decline and middle temporal gyrusis identified as a critical region of memory that changes in the early stage of AD, followed by progressive neocortical damage. Atrophy of middle temporal gyrus is now considered to be a valid diagnostic marker in the early stage of AD [21]. Our study showed that the minor allele (T) of rs7943454 increased the atrophy rate of right middle temporal gyrus in the one-year follow-up study and the C-allele remarkably prevented its atrophy. This result further proved that rs7943454 was associated with the onset risk of AD and *LUZP2* may be a new susceptibility gene of AD. However, we didn’t find any relation between rs7943454 and right middle temporal gyrus in subgroups due to the limited sample size.

The variation rs640476 is located on chromosome 5 q34, the intergenic region between *GABRB2* (gamma-aminobutyric acid type A receptor beta2 subunit) and *LOC285629* (also known as *LINC02159*, long intergenic non-protein coding RNA 2159). *GABRB2* is the gene coding β2 subunit of γ-aminobutyric acid receptor type A (GABAA receptor), which is the major mediator of fast inhibitory synaptic transmission in the central nervous system. Mutations in *GABRB2* genes have been reported to be associated with intellectual disability and epilepsy [22, 23]. A missense mutation in *GABRB2* was also reported to be associated with early myoclonic encephalopathy [24]. Mutations in *GABRB2* may reduce the expression of GABAA receptor and change the channel function, which could perturb GABA ergic inhibition in the brain. Disruption of excitatory-inhibitory (E/I) balance may be an important mechanism contributing to AD cognitive decline. Interestingly, despite vast neuronal loss in AD patients, GABA ergic neurons and receptors are relatively spared [25]. We have conducted a linkage disequilibrium analysis in order to annotate the identified functional variants. We found five loci which has strong linkage disequilibrium with rs7943454 (rs1509601: *r*^2^ = 0.81, D’ = 1; rs7927899: *r*^2^ = 0.81, D’ = 1; rs6484052: *r*^2^ = 0.99, D’ = 1; rs6484053: *r*^2^ = 0.8, D’ = 0.98; rs4922682: *r*^2^ = 0.91, D’ = 0.96). All these loci are also located in an intron region. We detected only one locus which has strong linkage disequilibrium with rs640476 (rs587875: *r*^2^ = 0.98, D’ = 1) and was also in an intergenic region.

Replication studies with independent, larger samples will be important to confirm these findings. In this study, we used a stringent MAF threshold (MAF > 0.20) and stringent Bonferroni corrections. These restrictions can improve statistical power to avoid false positive result but may miss less common SNPs. The modest number of subjects restricts stratified analyses for the three diagnostic groups. Besides, a two-year follow-up may be too short to observe the influence of rs7943454 on brain structure changes.

## Conclusions

In summary, we identified that two SNPs (rs7943454 in *LUZP2* and rs640476 near *GABRB2*) were associated with plasma NFL at suggestive levels. Rs7943454 in *LUZP2* was associated with the onset risk of AD and atrophy of right middle temporal gyrus in the whole cohort. Using endophenotype-based approach, we identified rs7943454 as a new AD risk locus.

## Additional file

Additional file 1: **Figure S1.** Plasma neurofilament light for AD diagnosis. Receiver operating cuves of logistic regression model are controlled for age at baseline, gender, educational level and APOE ε4 genotype. (PDF 6 kb)

## Abbreviations

AD: Alzheimer disease
ADNI: Alzheimer’s Disease Neuroimaging Initiative
ANOVA: One-way analysis of variance
*APOE*: Apolipoprotein E
Aβ: amyloid-β
CSF: Cerebrospinal fluid
*GABRB2*: Gamma-aminobutyric acid type A receptor beta2 subunit
GWAS: Genome-wide association study
ICV: Intracranial volume
IGAP: The International Genomics of Alzheimer’s Disease Project
*LUZP2*: Leucine zipper protein 2 gene
MAF: Minimum minor allele frequencies
MCI: Mild cognitive impairment
MRI: Magnetic resonance imaging
NFL: Neurofilament light
NFT: Neurofibrillary tangles
PET: Positron emission tomography
p-tau: Phosphorylated tau
t-tau: Total-tau
QC: Quality control
ROI: Regions of interest
SD: Standard deviations
SNPs: Single nucleotide polymorphisms
WAGR: Wilms tumor-Aniridia-Genitourinary anomalies-mental Retardation
