# Supplementary figures and images for: Genome-wide association study identifies two loci influencing plasma neurofilament light levels
Source: BMC Med Genomics. 2018 May 10;11:47. doi: 10.1186/s12920-018-0364-8 (PMC5946407; doi:10.1186/s12920-018-0364-8)

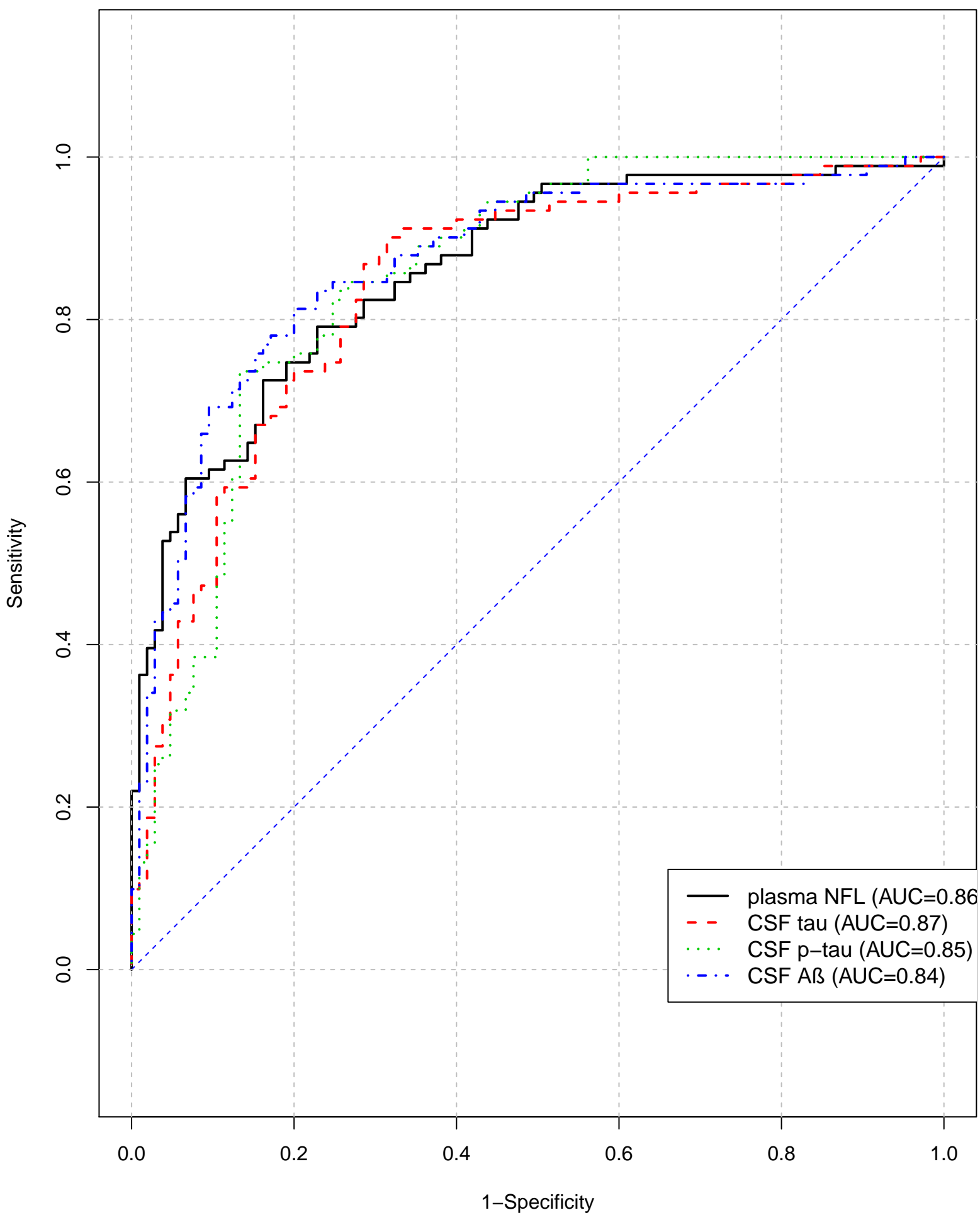

Supplement: Supplementary file 1 — Figure S1. Plasma neurofilament light for AD diagnosis. Receiver operating cuves of logistic regression model are controlled for age at baseline, gender, educational level and APOE ε4 genotype. (PDF 6 kb) [file 12920_2018_364_MOESM1_ESM.pdf]
